# Measuring thyroid-stimulating hormone receptor antibodies using the IMMULITE^®^ 2000 TSI assay is better than using the BRAHMS TRAK assay in an unselected clinical population, but both perform worse than expected: a real-world retrospective observational study

**DOI:** 10.1186/s13044-026-00296-5

**Published:** 2026-04-28

**Authors:** Sofia Manousou, Anders Olsson, Zoi Mamasoula, Mina Abdi Saran, Göran Oleröd, Helena Filipsson Nyström

**Affiliations:** 1 Department of Cardiology and Diabetes, Högsbo Hospital, Lilla Kapplandsgatan 10, Västra Frölunda, SE-421 37 Sweden; 2 Department of Internal Medicine and Clinical Nutrition, Institute of Medicine, University of Gothenburg, Box 428, Göteborg, SE-405 30 Sweden; 3 Wallenberg Centre for Molecular and Translational Medicine, Box 100, Göteborg, SE- 405 30 Sweden; 4 Department of Clinical Chemistry, Sahlgrenska University Hospital, Bruna Stråket 16, Göteborg, SE-413 45 Sweden; 5 Department of Endocrinology, Sahlgrenska University Hospital, Blå Stråket 5, Göteborg, SE-413 45 Sweden; 6 Gothenburg Centre for Person-Centred Care, Institute of Health and Care science, University of Gotheburg, Box 457, Göteborg, SE-405 30 Sweden

**Keywords:** Thyroid diagnostics and treatment, Endocrinology, Immunoassay, Immunoglobulin, Thyroid, KRYPTOR, Graves’

## Abstract

**Background:**

Graves’ hyperthyroidism is caused by stimulatory autoantibodies. Its diagnosis and monitoring are commonly based on measurement of thyrotropin receptor antibodies (TRAb) with unspecific immunoassays, that also detect neutral and blocking antibodies. TRAb analyzed using the Siemens IMMULITE^®^ 2000 TSI immunoassay (TRAb-IM), designed to target stimulatory immunoglobulins, represents a promising alternative. This study aimed to determine the clinical performance of TRAb-IM and the Thermo Fisher BRAHMS TRAK KRYPTOR immunoassay (TRAb-KR) in a real-world setting.

**Methods:**

Over 3 months, TRAb-IM was analyzed in samples collected to measure TRAb-KR after referral for thyrotoxicosis or at the time of discontinuation of antithyroid drugs (*n* = 168). Data on thyroid hormones, TRAb-KR, and date of start/discontinuation of antithyroid drugs was collected.

**Results:**

Agreement analysis for Graves’ disease diagnosis between the assays yielded a Gwet’s AC1 of 0.69 (95% confidence interval [CI] 0.51–0.86) for the samples collected after referral for thyrotoxicosis (*n* = 122). In this group, sensitivity (95% CI) for TRAb-IM and TRAb-KR was 97% (86–100) and 78% (62–90), respectively, in overt hyperthyroidism (*n* = 49), and 71% (42–92) and 43% (18–71), respectively, in subclinical hyperthyroidism (*n* = 46). Specificity was 100% (74–100) for both assays in overt hyperthyroidism, and 97% (84–100) and 100% (89–100), respectively, in subclinical hyperthyroidism. When TRAb-IM and TRAb-KR results were used as predictors for recurrence at the time of discontinuation of antithyroid drugs, the ROC AUC was 0.65 (95% CI 0.47–0.82; *p* = 0.07) and 0.57 (95% CI 0.41–0.73; *p* = 0.40), respectively.

**Conclusions:**

The TRAb-IM assay presented better clinical performance at both diagnosis of Graves’ disease and prediction of its recurrence compared to the TRAb-KR assay. Nonetheless, endocrinologists should be aware that both assays are weak in diagnostic of subclinical cases and in the prediction of recurrence, when used at the time of discontinuation of antithyroid drugs.

## Introduction

Graves’ disease (GD), one of the most common autoimmune diseases in Sweden [1], is caused by stimulatory autoantibodies targeting thyroid-stimulating hormone (TSH) receptors in the thyroid gland, resulting in hyperthyroidism. Today, the diagnosis of GD in Sweden is based on measuring TSH-receptor antibodies (TRAb) immunoassays that are generally not specific for stimulatory properties and detect also neutral and blocking antibodies. It is also expected that in approximately 5–10% of cases with GD, TRAb level is normal, and a thyroid scintigraphy or a thyroid ultrasonography is needed to discriminate GD from other types of hyperthyroidism [2–5]. TRAb is also used to monitor antithyroid drug (ATD) therapy, but the biological activity of TRAb is heterogeneous and may change during the course of the disease from stimulatory to blocking functional properties or vice versa [6, 7]. Neutral antibodies have also been detected in patients with GD [8]. TRAb measurement can therefore be misleading, both at diagnosis and during follow-up of GD [9]. On this ground, the European Thyroid Association suggests the use of highly sensitive cell-based bioassays in complex situations, because they exclusively differentiate between the stimulating and blocking antibodies [3].

While cell-based bioassays are preferred, the assays are often unavailable. Thus, the use of a bridge-based binding immunoassay, Siemens IMMULITE^®^ 2000 TSI immunoassay (TRAb-IM), designed to target stimulatory immunoglobulins, has emerged as an alternative to the unspecific TRAb immunoassays. TRAb-IM is biochemically validated [10–18] and may facilitate correct diagnosis [11–14, 16–18]. However, there have been reports indicating the assay to suffer from cross-interference of certain blocking TRAb to variable extent [19, 20]. The available data on TRAb-IM performance mainly derives from study populations, where subclinical cases are scarce or missing and therefore do not represent real-life conditions. In addition, how TRAb-IM can be used at follow-up has yet to be determined. A growing number of laboratories in Europe have replaced more unspecific TRAb immunoassays by TRAb-IM assay, whereas the current Swedish National Guideline only recommends TRAb-IM as an additional step in complex cases [4]. Notably, the use of “Graves’ Recurrent Events After Therapy” (GREAT) score [21], which is recommended in the Swedish National Guideline to predict the recurrence risk of GD after ATD treatment, was developed and validated using an unspecific TRAb assay. The GREAT score can be calculated at the time of GD diagnosis and is recorded on a 6-point scale (from 0 to 6 points) based on TRAb level (< 6, 6 to < 20, and ≥ 20 IU/L as 0, 1, and 2 points, respectively); age (≥ 40 or < 40 years as 0 or 1 point, respectively); goiter (not visible to slightly visible or clearly visible as 0 or 2 points, respectively); and free thyroxine (FT4) level (< 40 or ≥ 40 pmol/L as 0 or 1 point, respectively). Thus, 33% of the total score is derived from the TRAb result, but the corresponding TRAb-IM GREAT score cut-offs have not been established, making a GREAT score calculation impossible.

There are multiple assessments of the analytical performance of unspecific TRAb immunoassays and TRAb-IM [11, 12, 16, 17, 22–28]. The performance of the tested assays varies in the literature, depending on the chosen cut-off level, the manufacturer, as well as the use of a control population and its characteristics (i.e. healthy individuals, patients with other thyroid disease or with other non-thyroid disease). Generally, the sensitivity and specificity decrease when using other control populations than healthy individuals. In a real-world situation the diversity of patients tested is large and the assay must discriminate GD from other thyroid diseases. As real-world data was lacking at the planning stage of this study, the purpose was to determine whether the TRAb-IM immunoassay is more beneficial than unspecific TRAb immunoassays in the diagnosis and follow-up of GD in a random, unselected cohort at a secondary referral center in Sweden. To investigate the assays’ performance in a clinical context, they were mainly evaluated as dichotomous variables. For a clinician, it is crucial if the test is positive or not; a positive TRAb result is regarded as diagnostic for GD, whereas a negative value usually leads to further evaluation with thyroid scintigraphy or ultrasonography. The presence of antibody positivity during follow-up, has similarly important impact on the length of the ATD treatment. The specific aims were therefore: (1) to evaluate the sensitivity and specificity of TRAb-IM, TRAb by Thermo Fisher BRAHMS TRAK KRYPTOR immunoassay (TRAb-KR), and their combination for diagnosis; (2) to determine corresponding TRAb-IM cut-offs instead of TRAb-KR in the GREAT score; and (3) to determine the predictive potential of TRAb-IM and TRAb-KR for GD recurrence after discontinuation of ATD treatment. The clinically relevant question to be answered was whether unspecific TRAb immunoassays should be replaced by TRAb-IM or if these tools should be combined.

## Materials and methods

### Samples

**Fig. 1:**
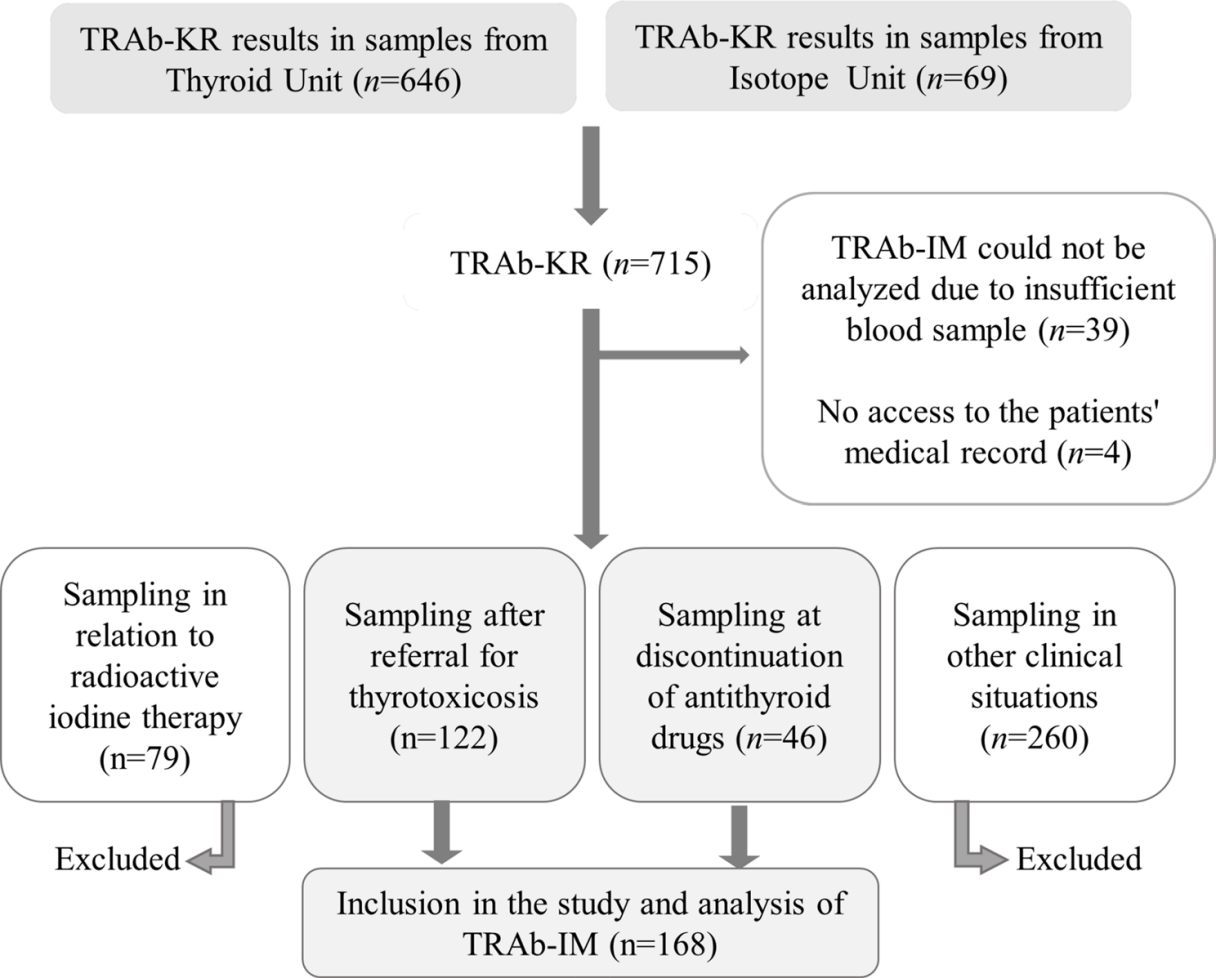
Flowchart illustrating the inclusion of samples for analysis with TRAb-IM in consecutive blood samples originally collected for clinical analysis with TRAb-KR. *Abbreviations*: TRAb-IM, Siemens IMMULITE^®^ 2000 TSI immunoassay; TRAb-KR, Thermo Fisher BRAHMS TRAK KRYPTOR immunoassay

Samples for TRAb-KR analysis collected from January to April 2019 at the Thyroid and Isotope Units, Sahlgrenska University Hospital (SU), Göteborg, Sweden were considered for inclusion in the study. The samples that were collected after referral for thyrotoxicosis (*n* = 122) or at ATD discontinuation (*n* = 46) were included in the study, as long as TRAb-IM could be analyzed. The group of samples collected at ATD discontinuation comprised specimens taken in euthyroidism just before or within 8 weeks after ATD discontinuation. Samples collected under other circumstances, were excluded (Fig. 1).

### Study design

In this investigator-initiated, real-world, retrospective study, the clinician had access only to TRAb-KR, and not to TRAb-IM. TRAb-KR was analyzed consecutively as part of clinical routine. The remaining serum volume of the samples was frozen and stored. TRAb-IM was analyzed in two batches within a 2-month period after the original TRAb-KR analysis. Data for age, gender, free triiodothyronine (FT3), FT4, TSH and TRAb-KR was collected from medical records. All samples taken after referral for thyrotoxicosis were collected before any ATD treatment, as ATD initiation is the responsibility of the endocrinologist in Sweden. The clinical diagnosis of GD registered in the patient file was based according to clinical practice in Sweden on: (1) TRAb-KR, (2) clinical assessment, and (3) thyroid scintigraphy in case of persistent hyperthyroidism combined with negative TRAb-KR. The thyroid scintigraphies were performed at the Department of Nuclear Medicine, SU, Göteborg and the clinician (endocrinologist) received both the image and an interpretation from the nuclear medicine expert, who – in line with common praxis– comment on the intensity of the thyroid gland even if the shape appears normal and the uptake homogenous. The sampling occasions were divided into those collected during overt hyperthyroidism (low TSH combined with FT4 and/or FT3 above the reference range) and subclinical hyperthyroidism (low TSH and normal FT4 and FT3). Subclinical hyperthyroidism was further divided into those with suppressed TSH (< 0.10 mIU/L) and those with low TSH (0.10–0.29 mIU/L).

Patient files were reviewed by two independent raters (ZM, HFN) to determine the final thyroid diagnosis, which could deviate from the clinical diagnosis recorded in the patients’ files, as the final diagnosis in the current study took into account data not available at the time of sampling such as the later natural clinical course and response to the therapy. In case of inconsistency, a third person (SM) reviewed the files and the case was discussed until consensus. The final diagnosis, as well as the GD recurrence rate were determined after a follow-up of 38–55 months from referral for thyrotoxicosis.

### Thyroid hormone assays

Thyroid hormones (TSH, FT4 and FT3) were analyzed by electrochemiluminescence methods using Roche Cobas 8000^®^ (Roche, Mannheim, Germany). The measuring uncertainty (given as coefficient of variation, CV) of the assays were: TSH < 4%CV at 0.8 mIU/L and < 3%CV at 10 mIU/L; FT4, < 5%CV at 16 pmol/L and < 6%CV at 32 pmol/L and FT3, < 6%CV at 14 pmol/L and < 5%CV at 5.8 pmol/L. The test results were evaluated in respect to the reference intervals given by the manufacturer, i.e. for TSH, FT4 and FT3, 0.30–4.2 mIU/L, 12–22 pmol/L and 3.1–6.8 pmol/L, respectively. The reference intervals were not stratified by sex or age, in accordance with general clinical practice.

### TSH-receptor autoantibody assays

TRAb-KR was analyzed with a chemiluminescence method using BRAHMS TRAK human KRYPTOR assay (Thermo Fisher Scientific, Hennigsdorf, Germany). TRAb-KR concentrations < 1.8 ΙU/L were considered negative (manufacturer’s cut-off) and measuring uncertainty at 3 and 10 IU/L was < 8%CV and < 5%CV, respectively. The TRAb-IM was performed with a bridge-based binding chemiluminescence method using Siemens IMMULITE^®^ 2000 TSI assay (Siemens Healthcare, Llanberis, UK). TRAb-IM concentrations < 0.55 IU/L were considered negative (manufacturer’s cut-off) and measuring uncertainty at 1 and 20 IU/L was < 5%CV and < 5%CV, respectively.

### Statistical methods

Sensitivity, specificity, and positive and negative predictive value of TRAb-KR and TRAb-IM were calculated after referral for thyrotoxicosis and at the time of ATD discontinuation to predict GD recurrence. A multivariable logistic regression model was fitted with GD as the binary outcome and with TRAb-KR and TRAb-IM as independent dichotomous variables to assess their combined ability to predict GD. The area under the receiver operating characteristic curve (ROC AUC) was calculated to assess the predictive performance. The degree of agreement between TRAb-KR and TRAb-IM was quantified using Spearman’s rank correlation when both variables were treated as continuous measures. When treated as dichotomous variables, agreement was assessed by evaluating the proportion of concordant results (i.e., when both were positive or both were negative) and by using Gwet’s AC1, where a value of 0.80 indicates substantial agreement [29]. To calculate the TRAb-IM cut-offs that correspond to the TRAb-KR cut-offs in the GREAT score, we used a Deming regression. The maximum value of Youden’s *J* statistic was used to define the best cut-off value of TRAb-KR or TRAb-IM to predict recurrence. This index can be represented graphically as the height above the chance line. To solve the problem of dependency between observations when more than one sample was derived from the same patient, a mixed model with random effect of patient was used to account for correlation between these measurements. Relative risk was calculated from a mixed model and compared to relative risk calculated when all observations were considered independent. As the results from the mixed model were similar to results from independent observations, all analyses were considered independent between observations. All analyses were performed using SAS^®^ v9.4 (SAS Institute, Cary, NC).

## Results

### Study population

The samples collected after referral for thyroxicosis (*n* = 122) were derived from 104 individuals (range: 22–86 years), including 84% females. The samples collected at discontinuation of ATD (*n* = 46) derived from 36 individuals (range: 23–87 years), including 83% females.

### TRAb-KR, TRAb-IM, and their combination TRAb-IM/TRAb-KR levels as diagnostic markers for GD

**Fig. 2:**
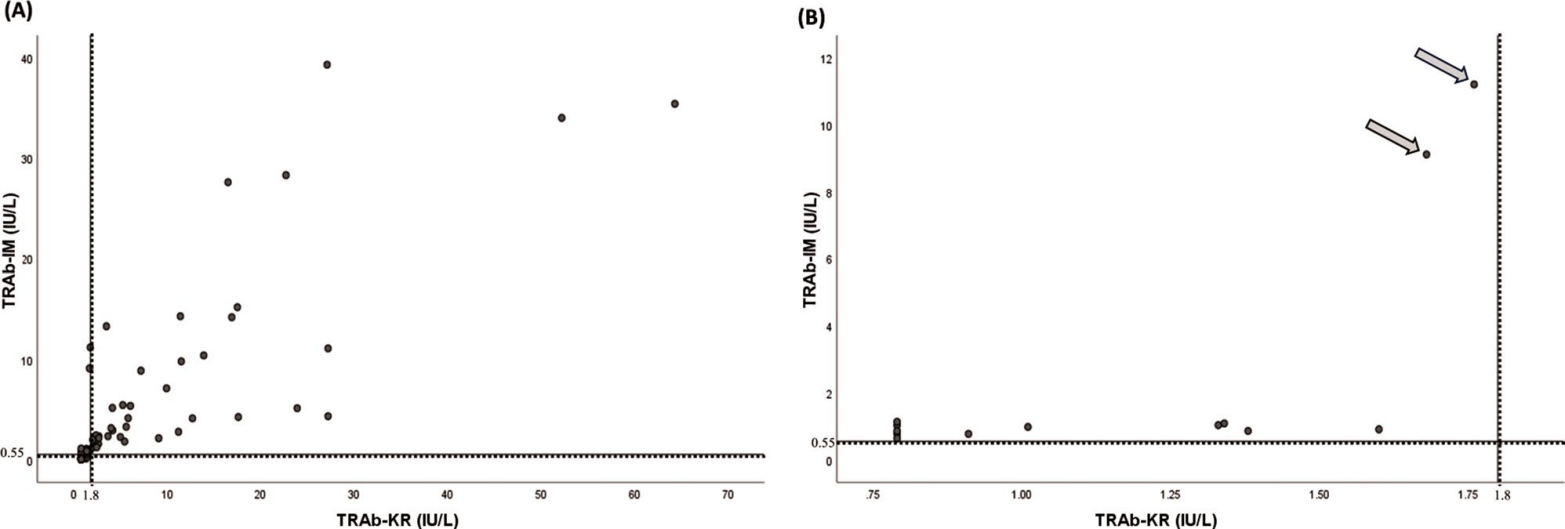
Scatter plot illustrating TRAb-KR and TRAb-IM values after referral for thyrotoxicosis: (**A**) the whole cohort (*n* = 122) and (**B**) discordant cases only (*n* = 13). Cut-off values for TRAb-KR (1.8 IU/L) and TRAb-IM (0.55 IU/L) are indicated by dotted lines. The two circles marked with arrows correspond to two samples from patient #1 in Table 1. *Abbreviations*: TRAb-IM, Siemens IMMULITE^®^ 2000 TSI immunoassay; TRAb-KR, Thermo Fisher BRAHMS TRAK KRYPTOR immunoassay

Of the samples collected after referral for thyrotoxicosis (*n* = 122), 13 samples (from 9 individuals) were discordant (i.e. positivity was noted in only one of the two assays) (Fig. 2) and the clinical information for these individuals is summarized in Table 1. The values noted with arrows in Fig. 2B correspond to patient #1 in Table 1, who was pregnant. Other samples that were collected from pregnant women (*n* = 10 samples from 6 individuals) after referral for thyrotoxicosis presented no discordance between the assays. A Spearman correlation of *ρ =* 0.92 was observed between the two assays (95% confidence interval [CI] 0.89–0.95, *p* < 0.001). A confusion matrix and the degree of agreement between the two assays as categorical variables are provided in Table 2; there were no cases of patients where both assays were falsely positive at diagnosis. In contrast, both analyses were negative in five samples (from 4 patients) where GD was finally diagnosed (Table 3).

**Table 1:** Characteristics of samples (*n* = 13, deriving from 9 individuals) collected after referral for thyrotoxicosis, where TRAb-KR and TRAb-IM were discordant

Patient	Age (y), sex	GD	FT4 (pmol/L)	FT3 (pmol/L)	TSH (mIU/L)	Thyroid function	TRAb-KR (IU/L)^a^	TRAb-IM (IU/L)^b^	Comment
#1	29, F	+	41	11	0.01	OH	1.68	9.11	OH with dry skin and itching 2 weeks prior to first sample collection. Positive pregnancy test so scintigraphy could not be performed. OH at pregnancy weeks 3, 5, and 19 with spontaneous normalization at week 25 without treatment. History: GD at the age of 15 y.
			32	–	0.01	OH	1.76	11.20	Assessment: hCG effect ruled out based on too early onset; thyroiditis and non-thyroid illness ruled out based on FT4/FT3; diagnosed as GD.
#2	84, F	–	19	8	0.01	OH	< 0.80	1.14	Ultrasound and scintigraphy indicated toxic nodular goiter.
#3	29, F	+	15	4	0.37	Normal	1.34	1.09	SH with TRAb-KR 2.0 IU/L at 5 weeks and SH with thyrotoxicosis symptoms (tremor, tachycardia, dyspnea) 2 weeks prior to sample collection. Scintigraphy indicated GD or thyroiditis under recovery. TSH under recovery at 1.2 mIU/L as highest, i.e. no hypothyroid phase observed. Assessment: in lack of hypothyroid phase, diagnosed as GD.
#4	68, F	+	19	–	0.01	SH	1.33	1.04	History of autoimmune thyroiditis treated with levothyroxine. Despite withdrawal of levothyroxine, thyrotoxic symptoms (tachycardia, sweating, gritty eye sensation, intolerance to heat), and TRAb-KR 1.76 IU/L prior to the current sampling. Scintigraphy indicated GD. After recovery, normal thyroid function without levothyroxine; FT4 15 pmol/L and TSH 3.8 mIU/L at 1.5 y after thyrotoxic symptom onset. Assessment: mild GD.
			18	6	0.01	SH	1.38	0.87	
#5	48, M	+	48	15	0.01	OH	< 0.80	1.03	Scintigraphy indicated GD.
			39	–	0.01	OH	< 0.80	0.87	
#6	71, F	+	34	12	0.01	OH	1.01	0.98	Scintigraphy indicated GD.
			21	7	0.01	OH	< 0.80	0.64	
#7	42, F	+	29	10	0.01	OH	1.60	0.91	Scintigraphy indicated GD.
#8	59 M	+	13	6	0.04	SH	0.91	0.78	Scintigraphy was inconclusive. Treated with ATDs. After ATD discontinuation, recurrence with OH and positive TRAb-KR. Assessment: GD
#9	33, F	+	101	42	0.01	OH	< 0.80	0.77	Scintigraphy indicated GD

^a^Reference: <1.8 IU/L. ^b^Reference: <0.55 IU/L. *Abbreviations*: ATD, antithyroid drug; F, female; FT4, free thyroxine; FT3, free triiodothyronine; GD, Graves’ disease; hCG, human chorionic gonadotropin; M, male; OH, overt hyperthyroidism; SH, subclinical hyperthyroidism with TSH < 0.1 mIU/L; TRAb-IM, Siemens IMMULITE^®^ 2000 TSI immunoassay; TRAb-KR, Thermo Fisher BRAHMS TRAK KRYPTOR immunoassay; TSH, thyroid-stimulating hormone; y, year

**Table 2:** Agreement between dichotomous TRAb-KR and TRAb-IM for GD diagnosis after referral for thyrotoxicosis

	Both assays positive	Both assays negative	TRAb-KR positive/ TRAb-IM negative	TRAb-KR negative/ TRAb-IM positive	Total	Agreement (95%CI)	Gwet’s AC1
GD present	40	5	0	12	57	0.79 (0.66–0.89)	0.69 (0.51–0.87)
GD absent	0	64	0	1	65	0.98 (0.92–1.00)	0.98 (0.95–1.00)
Total	40	69	0	13	122	0.89 (0.82–0.94)	0.80 (0.69–0.90)

Each row represents the confusion matrix of the two variables. *Abbreviations*: CI, confidence interval; GD, Graves’ disease; TRAb-IM, Siemens IMMULITE^®^ 2000 TSI immunoassay; TRAb-KR, Thermo Fisher BRAHMS TRAK KRYPTOR immunoassay

**Table 3:** Characteristics of samples (*n* = 5, deriving from 4 individuals) collected after referral for thyrotoxicosis where both assays were falsely negative

Patient	Age (y), sex	GD	FT4 (pmol/L)	FT3 (pmol/L)	TSH (mIU/L)	Thyroid function	TRAb-KR (IU/L)^a^	TRAb-IM (IU/L)^b^	Comment
#1	80, M	+	21	–	0.01	SH	< 0.80	0.09	Scintigraphy indicated GD.
#2	57, F	+	18	–	0.06	SH	< 0.80	0.20	Scintigraphy indicated GD. History of GD with positive TRAb-KR.
#3	53, F	+	14	5	0.12	SH	1.34	0.15	Scintigraphy indicated GD. Strong thyrotoxicosis symptoms (sweating, intolerance to heat, tachycardia, hunger, anxiety), which regressed totally after initiation of ATD therapy.
			16	5	0.07	SH	1.10	0.18	
#4	26, F	+	24	6	0.10	OH	< 0.80	0.09	Scintigraphy indicated GD.

^a^Reference: <1.8 IU/L.^b^Reference: <0.55 IU/L. *Abbreviations*: ATD, antithyroid drug; F, female; FT4, free thyroxine; FT3, free triiodothyronine; GD, Graves’ disease; M, male; OH, overt hyperthyroidism; SH, subclinical hyperthyroidism; TRAb-IM, Siemens IMMULITE^®^ 2000 TSI immunoassay; TRAb-KR, Thermo Fisher BRAHMS TRAK KRYPTOR immunoassay; TSH, thyroid-stimulating hormone; y, year

**Fig. 3:**
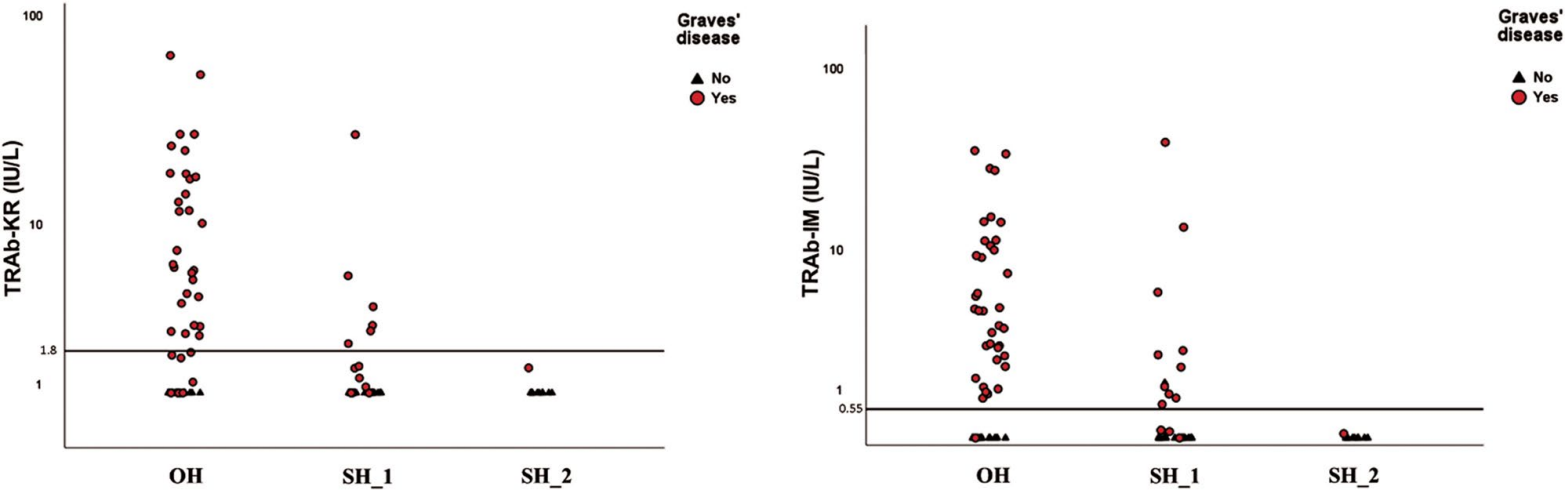
Distribution of TRAb-KR and TRAb-IM in samples collected after referral for thyrotoxicosis. The final diagnosis, as determined by the authors, is indicated by shape and color. Horizontal lines represent the cut-off values. Overt hyperthyroidism was defined as low TSH and high free thyroxine. Subclinical hyperthyroidism was defined as low TSH and normal free thyroxine. *Abbreviations*: OH, overt hyperthyroidism; SH_1, subclinical hyperthyroidism with thyroid-stimulating hormone (TSH) < 0.1 mIU/L; SH_2, subclinical hyperthyroidism with TSH 0.1–0.3 mIU/L; TRAb-IM, Siemens IMMULITE^®^ 2000 TSI immunoassay; TRAb-KR, Thermo Fisher BRAHMS TRAK KRYPTOR immunoassay

A confusion matrix, sensitivity, specificity, positive predictive value, and negative predictive value for TRAb-KR and TRAb-IM analyses are presented in Table 4 for the total number of samples and in subgroups separated by thyroid function test results. Distribution of TRAb-KR and TRAb-IM levels is shown in Fig. 3. The ROC AUCs are presented in Table 5 for all samples and separately for overt and subclinical hyperthyroidism. Including both TRAb-KR and TRAb-IM levels in the model (all samples) did not improve the results compared to using TRAb-IM levels alone (ROC AUC 0.95 [95% CI 0.92–0.99]). The TRAb-IM assay was positive in 13 more cases than the TRAb-KR assay, of which one was falsely positive (Table 1).

**Table 4:** Predictive potential of TRAb-KR and TRAb-IM for GD diagnosis after referral for thyrotoxicosis

	True positive	True negative	False positive	False negative		Percent (95% CI)			
						Sensitivity	Specificity	Positive predictive value	Negative predictive value
All samples (*n* = 122)									
TRAb-KR	40	65	0	17		70 (57–82)	100 (94–100)	100 (91–100)	79 (69–87)
TRAb-IM	52	64	1	5		91 (81–97)	98^a^ (91–100)	98 (90–100)	93 (84–98)
Overt hyperthyroidism (*n* = 49)									
TRAb-KR	29	12	0	8		78 (62–90)	100 (74–100)	100 (88–100)	60 (36–81)
TRAb-IM	36	12	0	1		97 (86–100)	100 (74–100)	100 (90–100)	92 (64–100)
Subclinical hyperthyroidism (*n* = 46)									
TRAb-KR	6	32	0	8		43 (18–71)	100 (89–100)	100 (54–100)	80 (64–91)
TRAb-IM	10	31	1	4		71 (42–92)	97 (84–100)	90 (59–100)	89 (73–97)
Subclinical hyperthyroidism with TSH < 0.1 mIU/L (*n* = 34)									
TRAb-KR	6	21	0	7		46 (19–75)	100 (84–100)	100 (54–100)	75 (55–89)
TRAb-IM	10	20	1	3		77 (46–95)	95 (76–100)	91 (59–100)	87 (66–97)
Subclinical hyperthyroidism with TSH 0.1–0.3 mIU/L (*n* = 12)									
TRAb-KR	0	11	0	1		0 (0–98)	100 (72–100)	–	92 (62–100)
TRAb-IM	0	11	0	1		0 (0–98)	100 (72–100)	–	92 (62–100)

^a^The specificity of 98% for TRAb-IM was explained by a sample from an 84-year-old patient with subclinical hyperthyroidism, undetectable TRAb-KR, TRAb-IM at 1.14 IU/L, and toxic multinodular goiter according to thyroid scintigraphy

In some cases (*n* = 27), the initial suspicion of hyperthyroidism could not be confirmed in the sampling that followed where antibodies were also analyzed. Overt hyperthyroidism was defined as low TSH and high free thyroxine. Subclinical hyperthyroidism was defined as low TSH and normal free thyroxine. *Abbreviations*: CI, confidence interval; GD, Graves’ disease; TRAb-IM, Siemens IMMULITE^®^ 2000 TSI immunoassay; TRAb-KR, Thermo Fisher BRAHMS TRAK KRYPTOR immunoassay; TSH, thyroid-stimulating hormone

**Table 5:** ROC AUCs for TRAb-KR and TRAb-IM for GD diagnosis after referral for thyrotoxicosis

	ROC AUC (95% CI)	*p*-Value
All samples (*n* = 122)		
TRAb-KR	0.85 (0.79–0.91)	< 0.001
TRAb-IM	0.95 (0.91–0.99)	< 0.001
Overt hyperthyroidism (*n* = 49)		
TRAb-KR	0.89 (0.82–0.96)	0.003
TRAb-IM	0.99 (0.96–1.00)	< 0.001
Subclinical hyperthyroidism (*n* = 46)		
TRAb-KR	0.69 (0.55–0.83)	0.026
TRAb-IM	0.83 (0.70–0.96)	< 0.001
Subclinical hyperthyroidism with TSH < 0.1 mIU/L (*n* = 34)		
TRAb-KR	0.71 (0.52–0.91)	0.082
TRAb-IM	0.90 (0.74–1.00)	0.004
Subclinical hyperthyroidism with TSH 0.1–0.3 mIU/L (*n* = 12)		
TRAb-KR	0.67 (0.46–0.87)	0.141
TRAb-IM	0.75 (0.53–0.97)	0.058

In some cases (*n* = 27), the initial suspicion of hyperthyroidism could not be confirmed in the sampling that followed where antibodies were also analyzed. Overt hyperthyroidism was defined as low TSH and high free thyroxine. Subclinical hyperthyroidism was defined as low TSH and normal free thyroxine. *Abbreviations*: CI, confidence interval; ROC AUC, area under the receiver-operating characteristic curve; TRAb-IM, Siemens IMMULITE^®^ 2000 TSI immunoassay; TRAb-KR, Thermo Fisher BRAHMS TRAK KRYPTOR immunoassay; TSH, thyroid-stimulating hormone

### TRAb-IM in relation to TRAb-KR levels in the GREAT score

From Deming regression we got the fitted regression line, representing the relation between TRAb-KR levels as independent variable and TRAb-IM levels as outcome. The equation of this fitted line was y = 0.437 + 0.709x with 95% CI for the intercept (–1.471 to 2.345) and for the slope (0.383–1.034), and where x and y represented TRAb-KR and TRAb-IM values respectively. By using the equation, we got a TRAb-IM value of 5 (95% CI 2–8) corresponding to the TRAb-KR cut-off of 6 and a TRAb-IM value of 15 (95% CI 7–23) corresponding to the TRAb-KR cut-off of 20 IU/L. The high variation makes conversion between TRAb-KR and TRAb-IM cut-off values for GREAT score uncertain.

### TRAb-KR and TRAb-IM levels as predictors of GD recurrence

Among patients with blood samples collected at discontinuation of ATD (*n* = 46), 37% (*n* = 17) relapsed. Sensitivity, specificity, positive predictive value, and negative predictive value for TRAb-KR and TRAb-IM when using the cut-offs that apply at diagnosis of GD are presented in Table 6. ROC AUCs showed that TRAb-KR had no ability to predict recurrence (0.57, 95% CI 0.41–0.73; *p* = 0.40), which is why no appropriate cut-off could be determined, whereas TRAb-IM showed a trend towards correlation with recurrence (0.65, 95% CI 0.47–0.82; *p* = 0.07), even though not reaching statistical significance. A cut-off of 0.81 IU/L was proposed for TRAb-IM to predict recurrence.

**Table 6:** TRAb-KR and TRAb-IM as predictors for GD recurrence at the time of ATD discontinuation

	Percent (95% CI)			
	Sensitivity	Specificity	Positive predictive value	Negative predictive value
Initial cut-off values				
TRAb-KR 1.8 IU/L	18 (4–43)	86 (68–96)	43 (10–82)	64 (47–79)
TRAb-IM 0.55 IU/L	59 (33–82)	72 (53–87)	56 (31–78)	75 (55–89)
Revised cut-off value				
TRAb-IM 0.81 IU/L	53 (28–77)	79 (60–92)	60 (32–84)	74 (55–88)

Initial cut-off values were the same as the ones used at diagnosis of hyperthyroidism. When the analytical method was proven to correlate to recurrence, a new cut-off was proposed based on ROC AUCs: TRAb-KR was not correlated while the recommended cut-off for TRAb-IM was 0.81 IU/L. *Abbreviations*: ATD, antithyroid drug; CI, confidence interval; ROC AUC, area under the receiver-operating characteristic curve; TRAb-IM, Siemens IMMULITE^®^ 2000 TSI immunoassay; TRAb-KR, Thermo Fisher BRAHMS TRAK KRYPTOR immunoassay

## Discussion

In this real-world study, both TRAb-IM and TRAb-KR performed worse than expected, but TRAb-IM was better than TRAb-KR as a marker for GD diagnosis and as predictor for GD recurrence at the time of ATD discontinuation. Composite TRAb-IM/TRAb-KR analysis had no additional value above TRAb-IM alone.

When investigating their diagnostic use, TRAb-IM and TRAb-KR showed sensitivities of 91% and 70%, respectively. The expected sensitivity for TRAb-IM and unspecific TRAb (including TRAb-KR) was assumed to be approximately 95% (or even higher for TRAb-IM), based on manufacturer’s claims and numerous studies [11, 12, 16, 17, 22, 23, 27, 28]. It is notable that only TRAb-IM in the subgroup of samples with overt hyperthyroidism in the current study showed comparable sensitivity (97%) to that in the cited literature. The expected sensitivity was based on studies using selected patients from biobanked material with known GD in combination with negative controls. This type of study design compared to studies including solely patients will in general give higher total specificity and sensitivity. This is due to the inherent nature of the calculation, given that the inclusion criteria favor both very healthy and more severe cases of disease. Furthermore, many studies present the sensitivity/specificity data based on optimal cut-offs determined by ROC analysis on the specific study material. We believe that most laboratories use the cut-offs proposed by the manufacturer, which are also the ground for the clinician’s decision. Additionally, the sensitivity for the assays increases when the calculation is based solely on samples with overt hyperthyroidism, which was not the case in the current study conducted at a secondary referral center. In Sweden, all patients with thyrotoxicosis are managed by an endocrinologist, meaning that patients with low TSH for any reason, and not only patients clearly eligible for thyroid treatment, are referred. Overall, the published sensitivity data is not easily transferred to real-world conditions.

There is to our knowledge only one study [25] which has investigated the diagnostic performance of TRAb-IM and the Roche Elecsys^®^ TRAb immunoassay by using the manufacturers’ cut-offs in a similar patient material as ours. That study, conducted in Canada, reported a sensitivity of 88% for TRAb-IM, which is in line with our results, whereas the reported sensitivity of 89% for the Roche Elecsys^®^ TRAb was much higher than for TRAb-KR in our study. The latter may be due to the Roche assay having a better performance than TRAb-KR. Two other studies which have compared TRAb-IM and TRAb-KR in a study population with solely individuals with thyroid disease, report higher sensitivity than the current study, i.e. 94% for TRAb-IM and 87–93% for TRAb-KR [24, 26]. However, these reports are on study populations most likely unrepresentative in relation to the Swedish referral conditions. In the study by Struja et al. [24] only patients with TSH < 0.01 mIU/L were included which at least in theory should reduce the number of subclinical cases, and Sccappaticcio et al. [26] used optimized cut-offs close to the functional sensitivity of the assays, indicating that a lower sensitivity could be expected if the manufacturer’s cut-off had been used. Thus, our study and representative published data indicate that the clinician should expect a sensitivity of only approximately 90% using TRAb-IM results and as low as 70% using TRAb-KR results. The clinician should be aware that antibodies targeting TSH receptor perform worse in subclinical cases and that it is difficult to differentiate an outlier with normal thyroid function, especially at diagnosis, when the clinical course is unknown.

We also report that TRAb-IM had a specificity of 98% and an overall clinical performance at diagnosis of GD that was better than for TRAb-KR. These results agree with some previous reports [11, 12, 14, 16–18, 23, 24, 26], but not with the recently published Canadian study [25]. The final diagnosis was determined within 4–7 weeks from the first clinical assessment in the Canadian study, in contrast to the long follow-up of 38–55 months in the current study, allowing important components of the clinical course to be considered by the independent raters. During these 3–4.5 years, some patients had developed thyroid-associated opthamopathy, had relapsed in typical GD with overt hyperthyroidism or had presented typical for thyroiditis clinical course. Some subclinical cases with diagnostic uncertainty could, therefore, be classified with high certainty to a final diagnosis. Even though TRAb-IM presented superiority compared to TRAb-KR in the majority of the discordant cases, it did present false positivity in one case (Table 1, patient #2), where imaging methods revealed toxic nodular goiter. If this patient had been falsely diagnosed with GD, the consequences would have varied depending on the choice of treatment. If thyroidectomy or radioactive iodine were chosen, the patient would have received adequate treatment despite a false initial diagnosis. In contrast, ATD treatment would have meant unnecessary use of ATD with follow-up visits for 1–1.5 year in the presence of persistent disease. This would have resulted in scintigraphy, which would have led to a delayed but correct diagnosis.

The hypothesis that a composite TRAb-IM/TRAb-KR score would be superior for GD detection was not confirmed, which was also supported by the high Spearman’s correlation between the two assays. This new information is relevant in the current context, in which the addition of TRAb-IM at secondary referral centers is being considered, while unspecific TRAb immunoassays remain the recommended standard in primary care prior to referral to an endocrinologist, due to broader availability [4].

When it comes to the assays’ use as prognostic factors for recurrency at the time of ATD discontinuation, our study also indicates a superiority for TRAb-IM, which is in line with previous studies [12, 30] but should be interpreted with respect to the limited sample size of this subgroup. It is important to note that assay cut-offs are defined exclusively for diagnostic purposes; the variability in cut-offs reported across studies evaluating the post-diagnosis period underscores the absence of a universally accepted standard. When using the same cut-off for TRAb-IM as the manufacturer recommends at diagnosis (i.e. <0.55 IU/L), the sensitivity and specificity of TRAb-IM to predict recurrence were 59% and 72%, respectively, compared to 18% and 86% for TRAb-KR. With a new proposed TRAb-IM cut-off of 0.81 IU/L, the sensitivity and specificity were 53% and 79%, respectively. Therefore, TRAb-IM presented better sensitivity than TRAb-KR regardless of the cut-off used for TRAb-IM. On the other hand, the specificity of TRAb-IM was not found to be better than TRAb-KR for any of the cut-offs used. Fontes and colleagues [31] proposed a higher cut-off for TRAb-IM at 1.11 IU/L in the assessment of recurrence based on a cohort of 92 patients. When using this cut-off to predict recurrence, they observed high sensitivity and specificity for TRAb-IM at 93% and 89%, respectively. The discrepancy of these results compared to ours, both in the level of the proposed cut-off and analytical performance achieved, may be due to the fact that Fontes and colleagues only included patients with overt hyperthyroidism at GD diagnosis. Subclinical hyperthyroidism should theoretically challenge assay performance equally when unspecific TRAb immunoassays and TRAb-IM are used as diagnostic tools and for the prediction of recurrence. The exclusion of patients with subclinical hyperthyroidism may also explain the higher relapse rate that Fontes and colleagues observed (49%) compared to that of our study population (37%).

If TRAb-IM would be used to predict recurrence at the time of GD diagnosis, we would need revised cut-offs for TRAb-IM in the GREAT score [21]. The current study could not provide such cut-offs due to the poor correlation between TRAb-KR and TRAb-IM. This poor correlation between the two assays is also shown by Scappaticcio et al. [26] and is theoretically supported by the known individual differences in expression of different types of antibodies (stimulatory, blocking and neutral). A study designed to define the TRAb-IM cut-offs in GREAT score is needed to obtain an answer on this clinically relevant question.

The current results suggest the more general use of the TRAb-IM assay in future guidelines. Unfortunately, the TRAb-IM assay –only provided on the IMMULITE^®^ instrument platform– is currently the only fully automated immunoassay on the market designed to target stimulatory immunoglobulins [17]. This complicates more general use and provides a challenge for more direct recommendations in general guidelines. Unspecific TRAb immunoassays are, on the other hand, available on several automated platforms, where at least the Roche Elecsys^®^ and BRAHMS KRYPTOR assays have comparable performance and cut-offs [32]. However, the TRAb-KR assay suffers from a generally high imprecision around the proposed cut-off, with a reported measurement uncertainty of 13% CV [32] and estimated to approximately 10%–15% CV in our laboratory. This yields an uncertainty of up to ± 0.5 IU/L around the cut-off and will affect the uncertainty of any sensitivity/specificity calculations of the assay. Therefore, the development of immunoassays selectively targeting stimulating TRAb on other instrument platforms is warranted.

An important strength of this study is that it assesses assay performance in both overt and subclinical hyperthyroidism, which better reflects clinical reality. The evaluation of the assays as diagnostic tools was performed separately for overt and subclinical hyperthyroidism, which allows a better understanding of the results and their relation to other findings in the literature. An additional strength is related to the way the final diagnosis was determined; even though a complement with ultrasound of the thyroid gland would have been ideal, it is important to note that the assessment was done by independent raters, after a 3–4.5 years’ follow-up, meaning that the natural clinical course and the response to the therapy were taken into consideration. The characteristics of all cases with discordance between the assays or with falsely negative results despite concordance are presented in detail, for reasons of transparency. The study is limited by the lack of a control group from the general population and the low sample size in the group of specimens collected at ATD discontinuation. The investigators could not, for practical reasons, be blind with respect to TRAb-KR assessment. Although this could introduce bias in favor of TRAb-KR, the results still indicate superiority for TRAb-IM.

In conclusion, the current results suggest more general use of the TRAb-IM assay in future GD guidelines, as it presents better clinical performance at both diagnosis of GD and prediction of its recurrence. Nonetheless, endocrinologists should be aware that both assays are uncertain in diagnostic of subclinical cases and in prediction of recurrence, when used at the time of discontinuation of antithyroid drugs.

## Data Availability

The data generated during this study cannot be made publicly available, due to privacy concerns. Access to the anonymized data will be considered upon request and subject to ethical approval.

## Abbreviations

ATD: Antithyroid Drug
CI: Confidence Interval
CV: Coefficient of Variation
FT3: Free Triiodothyronine
FT4: Free Thyroxine
GD: Graves’ Disease
GREAT: Graves’ Recurrent Events After Therapy
OH: Overt Hyperthyroidism
ROC AUC: Area under the Receiver-operating Characteristic Curve
SH_1: Subclinical Hyperthyroidism and TSH < 0.1 mIU/L
SH_2: Subclinical Hyperthyroidism and TSH 0.1–0.3 mIU/L
SU: Sahlgrenska University Hospital
TRAb: Thyrotropin Receptor Antibodies
TRAb-IM: Siemens IMMULITE^®^ 2000 TSI immunoassay
TRAb-KR: Thermo Fisher BRAHMS TRAK KRYPTOR immunoassay
TSH: Thyroid-stimulating Hormone, Thyrotropin

## References

[1] Abraham-Nordling M, Byström K, Törring O, et al. Incidence of hyperthyroidism in Sweden. Eur J Endocrinol. 2011;165(6):899–905. 10.1530/EJE-11-0548. 21908653 10.1530/EJE-11-0548

[2] Nyström HF, Jansson S, Berg G. Incidence rate and clinical features of hyperthyroidism in a long-term iodine sufficient area of Sweden (Gothenburg) 2003–2005. Clin Endocrinol (Oxf). 2013;78(5):768–76. 10.1159/000490384. 23421407 10.1111/cen.12060

[3] Kahaly GJ, Bartalena L, Hegedüs L, et al. 2018 European Thyroid Association Guideline for the Management of Graves’ Hyperthyroidism. Eur Thyroid J. 2018;7(4):167–86. 10.1159/000490384. 30283735 10.1159/000490384 PMC6140607

[4] Nyström HF. Nationellt vårdprogram för hypertyreos; 2022 [accessed 2025 Jan 7]. Available from: https://vardpersonal.1177.se/globalassets/nkk/nationell/media/dokument/kunskapsstod/vardprogram/nationellt-vardprogram-for-hypertyreos.pdf.

[5] Zöphel K, Roggenbuck D, Schott M. Clinical review about TRAb assay’s history. Autoimmun Rev. 2010;9(10):695–700. 10.1016/j.autrev.2010.05.021G. 20594972 10.1016/j.autrev.2010.05.021

[6] Kamath C, Adlan MA, Premawardhana LD. The role of thyrotrophin receptor antibody assays in Graves’ disease. J Thyroid Res. 2012;2012:525936. 10.1155/2012/525936. 22577596 10.1155/2012/525936 PMC3345237

[7] Michalek K, Morshed SA, Latif R, et al. TSH receptor autoantibodies. Autoimmun Rev. 2009;9(2):113–6. 10.1016/j.autrev.2009.03.012. 19332151 10.1016/j.autrev.2009.03.012 PMC3753030

[8] Morshed SA, Ando T, Latif R, et al. Neutral antibodies to the TSH receptor are present in Graves’ disease and regulate selective signaling cascades. Endocrinology. 2010;151(11):5537–49. 10.1210/en.2010-0424. 20844004 10.1210/en.2010-0424 PMC2954721

[9] Diana T, Krause J, Olivo PD, et al. Prevalence and clinical relevance of thyroid stimulating hormone receptor-blocking antibodies in autoimmune thyroid disease. Clin Exp Immunol. 2017;189(3):304–9. 10.1111/cei.12980. 28439882 10.1111/cei.12980 PMC5543506

[10] Emerson CH. Automated method for measuring TSIs using TSHR-binding properties has good sensitivity and specificity for Graves’ disease. Clin Thyroidol. 2016;28(8):235–7. 10.1089/ct.2016;28.235-237.

[11] Allelein S, Ehlers M, Goretzki S, et al. Clinical evaluation of the first automated assay for the detection of stimulating TSH receptor autoantibodies. Horm Metab Res. 2016;48(12):795–801. 10.1055/s-0042-121012. 27923250 10.1055/s-0042-121012

[12] Autilio C, Morelli R, Locantore P, et al. Stimulating TSH receptor autoantibodies immunoassay: analytical evaluation and clinical performance in Graves’ disease. Ann Clin Biochem. 2018;55(1):172–7. 10.1177/0004563217700655. 28388869 10.1177/0004563217700655

[13] Frank CU, Braeth S, Dietrich JW, et al. Bridge technology with TSH receptor chimera for sensitive direct detection of TSH receptor antibodies causing Graves’ disease: analytical and clinical evaluation. Horm Metab Res. 2015;47(12):880–8. 10.1055/s-0035-1554662. 26079838 10.1055/s-0035-1554662

[14] Kemble DJ, Jackson T, Morrison M, et al. Analytical and clinical validation of two commercially available immunoassays used in the detection of TSHR antibodies. J Appl Lab Med. 2017;2(3):345–55. 10.1373/jalm.2017.024067. 33636837 10.1373/jalm.2017.024067

[15] Lytton SD, Ponto KA, Kanitz M, et al. A novel thyroid stimulating immunoglobulin bioassay is a functional indicator of activity and severity of Graves’ orbitopathy. J Clin Endocrinol Metab. 2010;95(5):2123–31. 10.1210/jc.2009-2470. 20237164 10.1210/jc.2009-2470

[16] Liu K, Fu Y, Li T, et al. Clinical efficacy of thyroid-stimulating immunoglobulin detection for diagnosing Graves’ disease and predictors of responsiveness to methimazole. Clin Biochem. 2021;97:34–40. 10.1016/j.clinbiochem.2021.07.014. 34331946 10.1016/j.clinbiochem.2021.07.014

[17] Tozzoli R, D’Aurizio F, Villalta D, et al. Evaluation of the first fully automated immunoassay method for the measurement of stimulating TSH receptor autoantibodies in Graves’ disease. Clin Chem Lab Med. 2017;55(1):58–64. 10.1515/cclm-2016-0197. 27331310 10.1515/cclm-2016-0197

[18] Kim JJ, Jeong SH, Kim B, et al. Analytical and clinical performance of newly developed immunoassay for detecting thyroid-stimulating immunoglobulin, the Immulite TSI assay. Scand J Clin Lab Invest. 2019;79(6):443–8. 10.1080/00365513.2019.1658216. 31453725 10.1080/00365513.2019.1658216

[19] Diana T, Wuster C, Kanitz M, et al. Highly variable sensitivity of five binding and two bio-assays for TSH-receptor antibodies. J Endocrinol Invest. 2016;39(10):1159–65. 10.1007/s40618-016-0478-9. 27197966 10.1007/s40618-016-0478-9

[20] Allelein S, Diana T, Ehlers M, et al. Comparison of a Bridge Immunoassay with Two Bioassays for Thyrotropin Receptor Antibody Detection and Differentiation. Horm Metab Res. 2019;51(6):341–6. 10.1055/a-0914-0535. 31207654 10.1055/a-0914-0535

[21] Vos XG, Endert E, Zwinderman AH, et al. Predicting the risk of recurrence before the start of antithyroid drug therapy in patients with Graves’ hyperthyroidism. J Clin Endocrinol Metab. 2016;101(4):1381–9. 10.1210/jc.2015-3644. 26863422 10.1210/jc.2015-3644

[22] Xu S, Shao W, Wu Q, Zhu J, Pan B, Wang B, Guo W. Evaluation of the diagnostic performance of thyroid-stimulating immunoglobulin and thyrotropin receptor antibodies for Graves’ disease. J Clin Lab Anal. 2023;37(8):e24890. 10.1002/jcla.24890. 37161617 10.1002/jcla.24890 PMC10290220

[23] Thermo Fisher Scientific Inc. The gold standard in Graves’ disease diagnosis, 2017 [accessed 2024 Feb 27]. Available from: https://www.brahms.de/en-gb/news-media/download-center/48-brahms-trak-human-graves-disease/file.html

[24] Struja T, Jutzi R, Imahorn N, et al. Comparison of five TSH-receptor antibody assays in Graves’ disease: results from an observational pilot study. BMC Endocr Disord. 2019;19(1):38. 10.1186/s12902-019-0363-6. 31023276 10.1186/s12902-019-0363-6 PMC6482584

[25] Kline GA, Proctor DT, Moledina N, et al. Performance of the Siemens’ thyroid stimulating immunoglobulin assay in the diagnosis of hyperthyroidism: Prospective cohort study. Clin Biochem. 2025;137:110938. 10.1016/j.clinbiochem.2025.110938. 40286898 10.1016/j.clinbiochem.2025.110938

[26] Scappaticcio L, Trimboli P, Keller F, et al. Diagnostic testing for Graves’ or non-Graves’ hyperthyroidism: A comparison of two thyrotropin receptor antibody immunoassays with thyroid scintigraphy and ultrasonography. Clin Endocrinol (Oxf). 2020;92(2):169–78. 10.1111/cen.14130. 31742747 10.1111/cen.14130

[27] Cheng X, Chai X, Ma C, et al. Clinical diagnostic performance of a fully automated TSI immunoassay vs. that of an automated anti-TSHR immunoassay for Graves’ disease: a Chinese multicenter study. Endocrine. 2021;71(1):139–48. 10.1007/s12020-020-02386-2. 32562184 10.1007/s12020-020-02386-2

[28] Tong M, Ding J, Huang B, et al. Evaluation of the application of TSH receptor stimulating autoantibodies and the optimization of detection strategy in Graves’ disease. Clin Chim Acta. 2021;521:34–9. 10.1016/j.cca.2021.06.017. 34144042 10.1016/j.cca.2021.06.017

[29] Landis JR, Koch GG. The measurement of observer agreement for categorical data. Biometrics. 1977;33(1):159–74. 843571

[30] Peng R, Xie P, Jin Z, et al. Significance of thyroid stimulating immunoglobulin and thyrotropin receptor antibody in Graves’ disease. J Clin Endocrinol Metab. 2024;dgae892. 10.1210/clinem/dgae892. 10.1210/clinem/dgae892 39715350

[31] Fontes R, Negri MM, Marui S, et al. A higher cut-off for thyroid-stimulating immunoglobulin (TSI) could better predict relapse in Graves’ disease? J Clin Lab Res. 2021;3(2). 10.31579/2768-0487/035.

[32] Higgins V, Patel K, Kulasingam V, et al. Analytical performance evaluation of thyroid-stimulating hormone receptor antibody (TRAb) immunoassays. Clin Biochem. 2020;86:56–60. 10.1016/j.clinbiochem.2020.08.007. 32858059 10.1016/j.clinbiochem.2020.08.007

